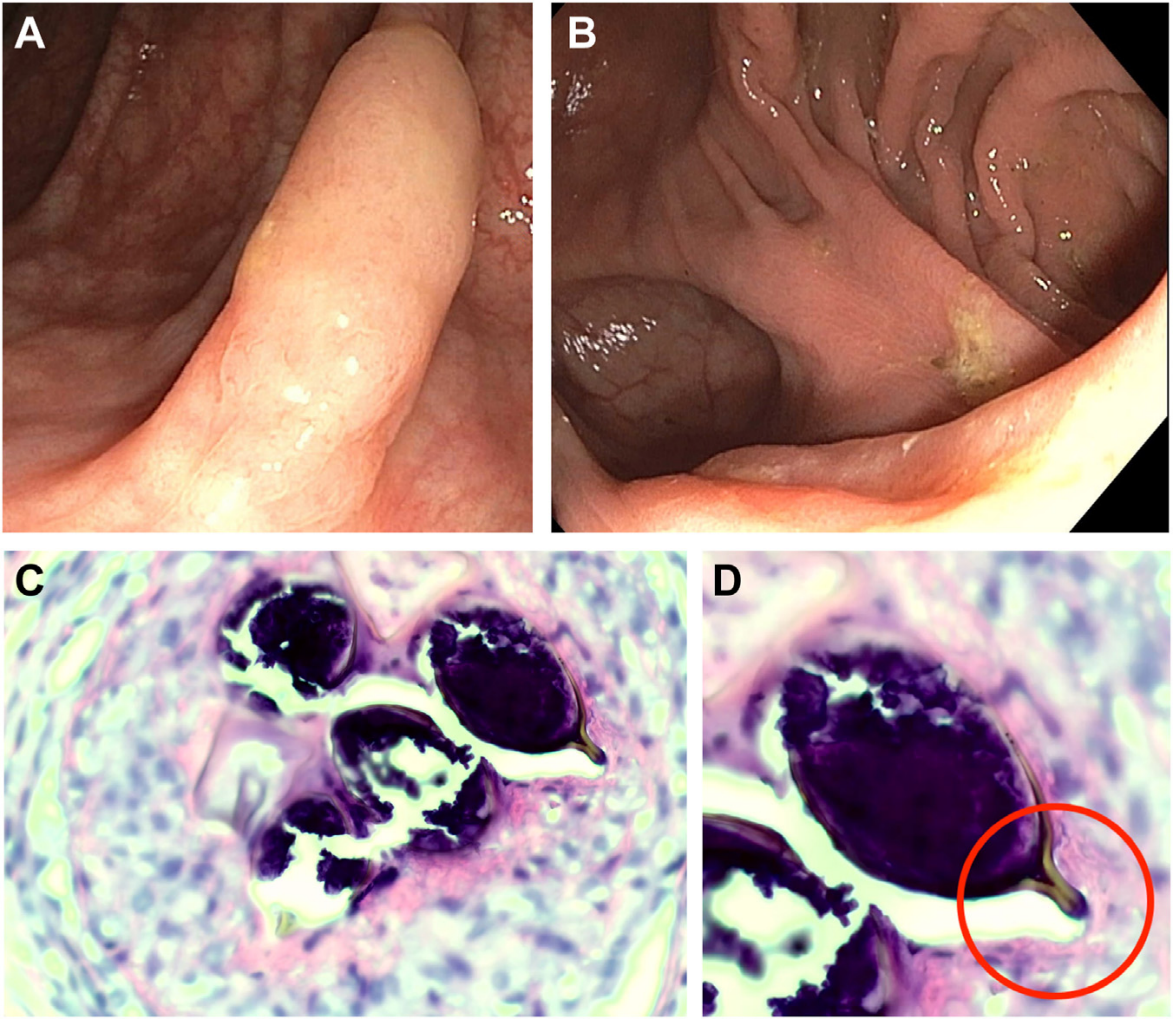# Chronic Schistosomiasis Infection Diagnosed via Polypectomy

**DOI:** 10.1016/j.gastha.2024.04.002

**Published:** 2024-04-15

**Authors:** Theodore W. James, Marek Skacel, Michael P. Sighinolfi

**Affiliations:** 1 Division of Gastroenterology, St. Joseph Health Care, Bangor, Maine; 2 Division of Surgical Pathology, Dahl-Chase Pathology Associates, Bangor, Maine

A 34-year-old woman underwent colonoscopy to evaluate 12 months of alternating diarrhea and constipation. The colonoscopy demonstrated normal-appearing mucosa throughout the colon and terminal ileum, with 3 polyps removed from the hepatic flexure and descending colon (Figure A). The colonic mucosa was normal (Figure B); biopsies were obtained throughout the colon to evaluate for microscopic colitis. Histopathology of the polyps was consistent with sessile serrated polyps; however, there was also a focal granulomatous reaction to possible parasitic eggs (Figure C). Microscopic colitis was absent on histology. Stool ova and parasite examinations 1 week post-colonoscopy and on 3 subsequent examinations were negative. The patient reported participating in a study abroad program 14 years prior in Tanzania, which included snorkeling in Lake Victoria. The patient denied any other significant travel or known exposures. A consultation obtained from the Centers for Disease Control and Prevention (CDC) confirmed the presence of a Schistosoma egg by the telltale terminal spine (Figure D) correlating with a diagnosis of *Schistosomiasis haematobium*. The patient was prescribed a single dose of praziquantel 40 milligrams/kilogram by mouth per CDC guidance. Following treatment, the patient denied a significant change in bowel habits, suggesting the possibility of concomitant irritable bowel syndrome.

**Figure:**